# What to wear? The influence of attire on the perceived professionalism of dentists and lawyers

**DOI:** 10.1111/jasp.12136

**Published:** 2014-08-06

**Authors:** Adrian Furnham, Pui Shuen Chan, Emma Wilson

**Affiliations:** University College London

## Abstract

Using a sample of 201 participants and a between-subjects design, the perceived professionalism—suitability, capability, ease to talk to and friendliness—of male and female dentists and lawyers in various attires was examined. Results showed an absolute preference for male dentists and lawyers in professional and formal attire, respectively. Male dentists and lawyers in professional and formal attire were further rated as more suitable, capable, easier to talk to, and friendlier than female professionals, and than those dressed in smart or casual attire. Results are discussed in terms of positive dental outcomes and legal representation. Limitations are considered.

## Introduction

Does how you dress influence how you are perceived, evaluated, and communicated with in the workplace? Sociologists and psychologists have long recognized the influence of one's appearance on important life experiences including interpersonal relationships and job-related successes (Bersheid & Gangestad, 1982; Gjerdingen & Simpson, 1989; Goffman, 1959; Rose, 1962). In particular, scholars have debated the formulaic nature of “dressing for success” (Cho & Grover, 1978; Levitt, 1981; Molloy, 1975, 1977; Solomon, 1986, 1987; Solomon & Douglas, 1983) and the study of attire in social organizations has a long history both within academic and applied communities (Becker, Greer, Hughes, & Strauss, 1961; Goffman, 1959; Simmel, 1971; Singer, Brush, & Lublin, 1965; Stone, 1962; Veblen, 1899). Organizational attire comprises the clothing (e.g., jacket, shirt, trousers) and artifacts (e.g., name tag, jewelry) that employees wear while at work (Rafaeli & Pratt, 1993).

The influence of attire on the perceived professionalism of dentists and lawyers in their respective job roles is the purpose of the present study. Over the last decade, there have been a number of studies on the assessment of doctors' clothing (Bond, Clamp, Gray, & van Dam, 2010; Cha, Hecht, Nelson, & Hopkins, 2004, Fischer, Hansen, Hunter, & Veloski, 2007; Gherardi, Cameron, West, & Crossley, 2009; Niederhauser, Turner, Chauhan, Magann, & Morrison, 2009) and almost nothing on any other profession. One exception was that on dental clinical attire (McKenna, Lillywhite, & Maini, 2007). In that study, participants were shown six pairs of photographs showing a young man and woman dressed in one of six different attires: modern dental tunic, pediatric surgical scrubs, plain surgical scrubs, traditional dental tunic, smart dress with white coat, and casual dress. There was a very strong preference for the traditional dental tunic with no one favoring casual dress. They also liked their dentist to wear name badges, safety glasses, and face masks.

The present study also focused on dental attire, using a broader range of styles that vary on the formality scale, but also investigates the reactions to lawyers wearing different attires. All the studies in this area have focused on those in the medical world, but reactions to other professionals are inevitably influenced by the particular clothes worn. Further, the formal–informal (smart–casual) nature of clothing may well have a different effect on male vs. female wearers. We will also look at gender effects in this study.

Professionalism can be described as a function of clinical skill, engagement and competence, or “an image, which promotes a successful relationship with patients” (Brosky, Keefer, Hodges, Pesun, & Cook, 2003). Across cultures and time periods, many professions have become identifiable by their uniforms including the police, nursing staff, and barristers. Professional dress codes are a set of standards that serve two functions—to provide employees with guidelines about what is appropriate to wear for work; and to provide a common in-group identity that separates them from other professions. In return, members of the profession are expected to act in a certain way and possess certain attributes, often in accordance with a code of conduct (Kalisch & Kalisch, 1985). Suitable work attire varies between industries and ranges from service uniforms, formal and business smart apparel, and casual attire. The formality of the workplace dress code is normally determined by the amount of interaction employees have with clients.

The uniforms of service workers have historically been worn to reflect the goals of the profession (e.g., sterility, fire resistance) while also being suitable for dealing with their client base. Similar to the majority of professions, the prescribed medical attire is not static. While the importance of physician attire has been traced back to Hippocrates (1923), the white coat has been the accepted medical symbol for the profession in the Western world for over 100 years (Blumhagen, 1979). Patients show a well-replicated preference for the familiar white coat and name tag (Colt & Solot, 1989; Douse, Derrett-Smith, Dheda, & Dilworth, 2004; Gjerdingen, Simpson, & Titus, 1987; Gonzalez Del Rey & Paul, 1995; Gooden, Smith, Tattersall, & Stockler, 2001; Harnett, 2001; Ikusaka et al., 1999; Matsui, Cho, & Rieder, 1998; Rehman, Nietert, Cope, & Kilpatrick, 2005) because of its automatic impressions of cleanliness, competence, and professionalism (Barrett & Booth, 1994; Becker et al., 1961; Hennessy, Harrison, & Aitkenhead, 1993; McKinstry & Wang, 1991; Taylor, 1987). However, the medical uniform has moved away from the white coat in the last decade, arguably because of concerns of spread of infection. Most noticeably, attire has gone from that considered unique to the profession to apparel that reflects a general societal shift toward comfort and informality (LaSala & Nelson, 2005).

Several studies have investigated the influence of physician attire on patient perceptions. Results suggest a clear pattern. A recent study conducted on a large patient sample investigated patient attitudes to doctors in different attires, varying in formality (Gherardi et al., 2009). Findings revealed that patients had most confidence in doctors wearing the symbolic white coat, followed by doctors in a long-sleeved shirt, tie, and tailored trousers (male) or long-sleeved shirt and knee-length skirt (female). This is likely due to patient exposure to this dress style. Casual attire was the least confidence inspiring because of its unkempt appearance. Other studies have shown a preference for formal dress (e.g., suit and tie for male physicians; blouse and skirt/tailored trousers for female physicians with minimum make-up and jewelry) rather than casual attire (e.g., jeans, t-shirt) (Gjerdingen et al., 1987; Gonzalez Del Rey & Paul, 1995; McKinstry & Wang, 1991; Swift, Zachariah, & Casy, 2000). Less formal attire conveys compassion, friendliness, and approachability in the physician (Gledhill, Warner, & King, 1997), but also incompetence and a failure to inspire patient confidence (Gherardi et al., 2009). Taken together, research on the influence of physician attire on patient perceptions generally finds that uniforms and formal attire generate authority and status; while casual attire, approachability, and patient disclosure.

Similar dress code shifts are evident in business. However, in contrast to service workers, business attire is not a uniform and the acceptability of work attire is more loosely defined, differs widely between organizations, and invariably causes confusion. The introduction of “Casual Friday's” is itself an indicator that dress codes have generally softened across the business community. In addition the advent of midpoints on the formality scale, including “Business Casual” and “Smart Casual,” has made dressing for success all the more difficult—arguably more so for women. In some traditional industries, including law and finance, a formal dark suit is generally accepted for both men and women. In other industries, less formal apparel such as jeans and a t-shirt are sometimes deemed appropriate as part of the business casual attire. Smart attire is more loosely defined, but generally translates as tailored trousers, long-sleeve shirt (tie optional), belt, and leather shoes for men, and typically for women, tailored trousers or a mid- to full-length skirt, belt, jacket, and flat or mid-heel shoes that coordinate with the outfit. Formality however is subjective and without a strict corporate dress code, the definitions mentioned earlier are not recognized in the workplace.

Research has generally concluded that formal attire conveys power and status across a number of contexts (Fortenberry, MacLean, Morris, & O'Connell, 1978; Kwon & Johnson, 1998). However, its influence on other important traits including social likeability (e.g., friendliness) is less clear. Dress is a form of communication (Lurie, 1981) and given its nonverbal influence on relationship formation, research has begun to investigate the influence of a societal shift on nonverbal communication and the interaction between professionals and those who choose to seek their expertise.

Competence, confidence, and credibility are judged in the first 12 seconds of an interaction, which is, at least in part, influenced by the clothes one is adorning (Bixler & Scherrer Dugan, 2000). In turn, such covert judgments are identified as influencing overt behavior change. The literature to date on the associations between perceived professionalism and attire has largely focused on clinical professions, namely medicine. While physician appearance may not be the most important aspect of the physician–patient relationship, it does play an important role and can influence willingness to share personal information, adherence to treatment regimen, and likelihood of attending future appointments. Taken together, the appearance of healthcare professionals and the apparel they wear during initial patient interactions can influence all aspects that are entailed in the long-term relationship between the two parties. In addition, there is considerable literature to suggest that, when compared with their male counterparts, female professionals are perceived as less competent, agentic, and status-driven. It is because of this incongruence between gender-typed female characteristics and those required for the job that women face additional pressure in their perceived professionalism over and above what they choose to wear to work (Eagly & Koenig, 2008; Eagly & Wood, 2000).

The various studies on ratings of professionals' attire suggests there are around four or five options open to them; either casual or “smart” general clothes that are fashionable at the time, or a variety of outfits (traditional, modern) associated with that profession, which may involve specific tunics, coats, or technical equipment (i.e., a stethoscope). This study presents participants with five options showing men and women wearing outfits that represent the options mentioned earlier.

The present study aimed to extend the findings of previous research by assessing the influence of attire on the perceived professionalism of dentists and (nonclinical) lawyers—two professions that involve limited professional-client interface and professions in which first impressions have an enduring impact on personal (health/legal) outcomes. The following hypotheses will be tested:

*Hypothesis 1a.* There will be an absolute preference for male dentists and lawyers (*Hypothesis 1b.* in professional/formal attire) over their respective female counterparts, irrespective of participant gender.

*Hypothesis 2.* Dentists dressed in professional, and lawyers in formal dress, will be perceived as the most suitable and capable in their respective professions. Professionals casually dressed in both groups will be rated the least suitable and least capable.

*Hypothesis 3.* Casual attire will be perceived as the most friendly and as easier to talk with for both professions while professional and formal apparel will be rated the least friendly and least easy to talk to for dentists and lawyers, respectively.

## Method

### Participants

In total, 201 participants were recruited in this study. Of these, 91 were male and 110 were female. Their age ranged from 18 to 60 years, with a mean of 24.59 years (standard deviation = 9.3). The majority had attained an undergraduate level qualification (71.6%) while 9% had received A-levels or equivalent, and 19.4% had continued onto postgraduate studies.

### Measures

Participants were shown a total of 20 photographs (10 dentists and 10 lawyers), adopted from earlier research undertaken by Barrett and Booth (1994) and Gledhill et al. (1997), respectively. Different studies have used very different photographs (Bond et al., 2010), some attempting to obscure the face of the person (Cha et al., 2004). Target gender was counterbalanced across occupations, each comprising five male and five female professionals in various attires, varying in formality.

**Figure 1:**
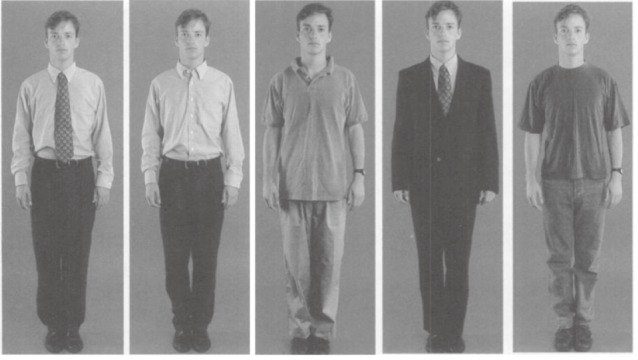
Attires for male lawyers.

**Figure 2:**
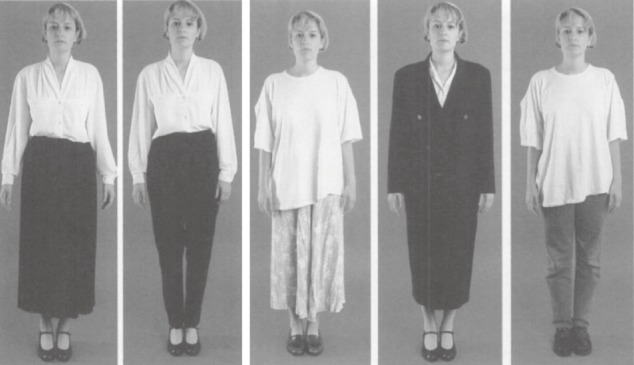
Attires for female lawyers.

**Figure 3:**
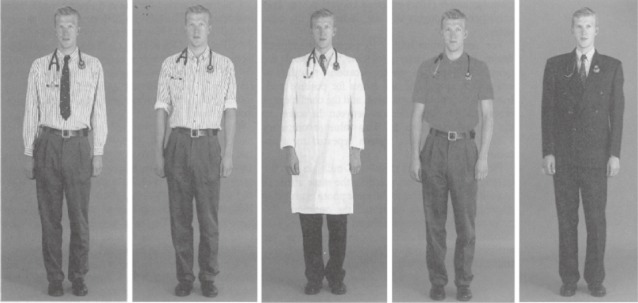
Attires for male dentists.

**Figure 4:**
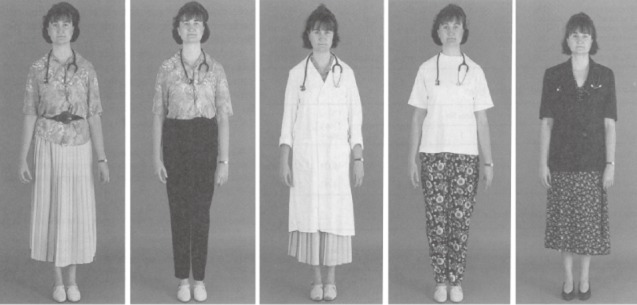
Attires for female dentists.

All of the photographs were color, full-figure shots with a plain background. Each depicted the same male or female model, possessing a neutral facial expression and dressed in one of five different attires: Dress for the male lawyer consisted of (1) a long-sleeved shirt, tie, and trousers; (2) a long-sleeved shirt and trousers without a tie (*smart dressing style*); (3) casual trousers and casual top; (4) suit and a tie (*formal attire*); and (5) jeans and a t-shirt (*casual attire*). Dress for the female lawyer consisted of (1) a blouse and skirt; (2) a blouse and trousers (*smart dressing style*); (3) a casual skirt and t-shirt; (4) a suit (*formal attire*); and (5) jeans and a t-shirt (*casual attire*; see Figures 1 & 2). The male model in the dentist condition was presented in the same attires as those in the male lawyer condition, except for photo shot 3, wherein casual top and trousers was replaced with a shirt, tie, trousers, and white coat (*professional attire*). The female model in the dentist condition wore the same attires as those presented in the female lawyer condition except for photo shot 3 in which the model wore a white coat in addition to the casual skirt and t-shirt (*professional attire*). Photo shot 4 shows the only form of casual attire presented in the dentist condition, for both the male and female model (see Figures 3 & 4).

Across conditions, photos 1 and 2 depict a smart dressing style, and photos 5 (dentists) and 4 (lawyers) illustrate formal dressing. Photo 4 in the dentist condition and 3 and 5 in the lawyer condition are referred to as casual dressing style. The additional photo in the dentist condition (photo 3) illustrates professional attire.

A problem that has to be faced by all researchers creating or choosing stimuli for these studies are possible confounds (e.g., age, attractiveness, fashionability of the clothing). Most studies made little attempt to ensure the “models” of different sex were of equivalent age, attractiveness, and perceived competency. Further the fashions in casual attire change more than in “smart” professional attire. We were conscious of this problem and hence conducted a pilot of the stimuli we planned to use. In the pilot phase, we asked 20 students to rate the four models on age and attractiveness. There was no significant difference on age and all models were thought of to be late 20s and early 30s. There was a marginally significant effect for “physical attractiveness” where the female dentist model was thought of as slightly (*p* = .05) less attractive than the other three models.

### Procedure

The majority of participants were approached in a variety of public settings including libraries, coffee bars and railway stations. A small number of participants were recruited through personal contacts. Participants were initially presented with the complete set of photographs for each condition and asked to provide their absolute preference, indicating how they would ideally like their dentist/lawyer to dress for work. In each condition, participants were then shown the photographs in a random order and asked to provide ratings concerning their perceived (1) suitability of the attire for the profession; (2) capability of the model; (3) ease of talking with the model; and (4) friendliness of the model.

Ratings were anchored by 1 (*Least Suitable/Capable/Easy to talk to/Friendly*) and 8 (*Most Suitable/Capable/Easy to talk to/Friendly*). Participants were asked to provide their ratings as quickly as possible, however no time limit was set.

Lastly, participants provided demographic information including gender, age and educational attainment.

## Results

### Absolute preference for professional attire

In order to investigate participant's absolute preference for dentists and lawyers, chi-squared statistic was performed. Results indicate that male and female participants did not significantly differ in their absolute preference, both showing a preference for the male dentist wearing the professional white coat: χ^2^ (7) = 6.74, *p* > .05. Similarly, there were nonsignificant gender differences in ratings for lawyers with both male and female participants showing a preference for the male lawyer wearing the formal dark suit: χ^2^ (7) = 7.03, *p* > .05. Thus, a gender bias for male professionals was found for both dentists and lawyers.

Table 1 breaks down participant absolute preferences for dentists and lawyers, split by target gender. Results suggest that the professional white coat rated more often as the preferred attire for men in dentistry. The majority of participants preferred the formal dark suit as the best attire for male and female lawyers.

**Table 1:** Participant Absolute Preferences for Male and Female Professionals in Various Attires

	Number (%) of participants rating best attire for			
	
	Dentist		Lawyer	
		
	Male	Female	Male	Female
D/L: Casual/Casual	13 (6.5)	20 (10.0)	2 (2.2)	1 (.5)
D/L: Smart/Casual	10 (5.0)	0 (.0)	0 (.0)	0 (.0)
D/L: Smart/Smart	37 (18.4)	0 (.0)	15 (7.5)	9 (4.5)
D/L: Formal/Smart	3 (1.5)	1 (5.0)	2 (1.0)	15 (7.5)
D/L: Professional/Formal	105 (52.2)	12 (6.0)	124 (61.7)	33 (16.4)

*Note.* D = dentists; L = lawyers.

Four 2 (Target sex) × 5 (Attire) repeated measures analysis of variance (ANOVA) with post hoc comparisons (Bonferroni corrected) were calculated for each profession to assess the perceived suitability, capability, ease to talk to, and friendliness of the dentist and lawyer models with respect to their roles. Target gender and the five attires (dentists: smart, smart, professional, casual, formal; lawyers: casual, casual, smart, smart, formal) acted as the within-subjects variables. Table 2 shows mean ratings, *F* ratios (for style) and partial eta-square values across the five attires; and the mean scores across attires, separated by target gender. The Attire × Target gender interactions found in each analysis revealed that male and female professionals, while adorning the same attire, received differential professionalism scores.

**Table 2:** Mean Scores (*SE*), *F* Values and Partial Eta-Square for Professionalism Traits across Attires for Dentists and Lawyers; Split by Target Gender

		Attire														
		
		Casual (trousers and top)		Smart 1 (tie/skirt)			Smart 2 (no tie/trousers)			Formal (dark suit)			Professional (white coat)			
Dentist		X (*SE*)		X (*SE*)			X (*SE*)			X (*SE*)			X (*SE*)		*F*	η^2^p
	Suitability	4.24 (.11)		3.96 (.09)		**<**	4.44 (.10)		**>**	3.33 (.10)		**<**	6.53 (.10)		193.47**	.49
		*M*	*F*	*M*	*F*		*M*	*F*		*M*	*F*		*M*	*F*		
		4.49 (.13)	3.99 (.14)^a^	5.11 (.13)	2.83 (.11)^a^		5.43 (.12)	3.46 (.12)^a^		3.58 (.13)	3.07 (.12)^a^		6.87 (.10)	6.20 (.12)^a^		
	Capability	4.49 (.11)		4.46 (.10)			4.57 (.10)		**>**	3.88 (.11)		**<**	6.53 (.10)		159.28**	.45
		*M*	*F*	*M*	*F*		*M*	*F*		*M*	*F*		*M*	*F*		
		4.93 (.13)	4.04 (.14)^a^	5.67 (.11)	3.24 (.12)^a^		5.50 (.12)	3.65 (.12)^a^		4.33 (.14)	3.42 (.13)^a^		7.00 (.10)	6.08 (.12)^a^		
	Ease of talking	4.74 (.10)		4.72 (.09)			4.79 (.09)		**>**	3.71 (.08)		**<**	5.42 (.11)		69.67**	.26
		*M*	*F*	*M*	*F*		*M*	*F*		*M*	*F*		*M*	*F*		
		5.23 (.12)	4.26 (.12)^a^	5.25 (.10)	4.21 (.13)^a^		5.48 (.11)	4.12 (.12)^a^		4.15 (.11)	3.27 (.11)^a^		5.68 (.11)	5.18 (.12)^a^		
	Friendliness	4.89 (.10)		4.77 (.09)			4.80 (.09)		**>**	3.66 (.09)		**<**	5.28 (.10)		69.33**	.26
		*M*	*F*	*M*	*F*		*M*	*F*		*M*	*F*		*M*	*F*		
		5.43 (.12)	4.36 (.12)^a^	5.19 (.10)	4.38 (.13)^a^		5.46 (.11)	4.17 (.12)^a^		4.08 (.11)	3.25 (.11)^a^		5.56 (.11)	5.04 (.12)^a^		

		Casual 1 (trousers/ skirt & top)		Casual 2 (jeans & t-shirt)			Smart 1 (tie/skirt)			Smart 2 (no tie/trousers)			Formal (dark suit)			
Lawyer		X (*SE*)		X (*SE*)			X (*SE*)			X (*SE*)			X (*SE*)		*F*	η^2^p
	Suitability	1.98 (.08)		1.86 (.08)		**<**	5.84 (.09)		**>**	5.42 (.10)		**<**	6.74 (.08)		109.55**	.85
		*M*	*F*	*M*	*F*		*M*	*F*		*M*	*F*		*M*	*F*		
		1.92 (.10)	1.99 (.09)	1.97 (.09)	1.71 (.08)^a^		6.47 (.10)	5.23 (.12)^a^		5.29 (.12)	5.59 (.11)		7.37 (.08)	6.12 (.12)^a^		
	Capability	2.40 (.09)		2.26 (.10)		**<**	5.85 (.09)		**>**	5.43 (.10)		**<**	6.70 (.08)		859.77**	.81
		*M*	*F*	*M*	*F*		*M*	*F*		*M*	*F*		*M*	*F*		
		2.34 (.11)	2.42 (.11)	2.40 (.11)	2.09 (.11)^a^		6.43 (.10)	5.29 (.11)^a^		5.25 (.12)	5.65 (.11)^a^		7.25 (.08)	6.15 (.11)^a^		
	Ease of talking	4.21 (.12)		4.10 (.13)		**<**	4.96 (.08)			5.04 (.09)			5.15 (.09)		35.01**	.15
		*M*	*F*	*M*	*F*		*M*	*F*		*M*	*F*		*M*	*F*		
		4.22 (.13)	4.21 (.14)	4.25 (.14)	3.95 (.14)^a^		5.40 (.09)	4.54 (.10)^a^		5.13 (.10)	4.97 (.10)		5.68 (.10)	4.63 (.10)^a^		
	Friendliness	4.24 (.12)		4.30 (.13)		**<**	4.86 (.08)			4.89 (.09)			4.98 (.09)		16.00**	.07
		*M*	*F*	*M*	*F*		*M*	*F*		*M*	*F*		*M*	*F*		
		4.22 (.14)	4.26 (.14)	4.54 (.14)	4.03 (.15)^a^		5.25 (.09)	4.49 (.11)^a^		4.96 (.10)	4.85 (.10)		5.51 (.11)	4.46 (.10)^a^

*Notes.* *M* = male target; *F* = female target.

* *p < .001.

< and > = significant comparisons between attires where *p* < .05.

a Significant target gender differences in professionalism rating where *p* < .01.

The analysis was then run on each of the four professionalism ratings. In each set of subsequent analyses, we looked at sex and attire differences in the two professionals separately.

### Attire and suitability

Question A assessed the suitability of the attires for the models respective profession (dentist or lawyer). Mauchley's test of sphericity yielded a significant effect of attire, D: χ^2^(9) = 151.64, *p* < .001; L: χ^2^ (9) = 245.07, *p* < .001; and a significant interaction of Gender × Attire, D: χ^2^(9) = 107.57, *p* < .001; L: χ^2^ (9) = 71.41, *p* < .001.

#### Dentists

All main effects were significant. Men (*M* = 5.10, standard error [*SE*] = .08) were considered more suitable for dentistry than women (*M* = 3.90, *SE* = .07), *F*(1,199) = 202.21, *p* < .001, η^2^p = .50. To determine which attire ratings differed significantly from the others, post hoc Bonferroni comparisons for the main effect of attire, *F*(4, 796) = 193.47, *p* < .001, η^2^p = .49, revealed that the professional white coat (*M* = 6.53, *SE* = .10) was rated significantly more suitable for dentists than either of the two smart attires (1: *M* = 3.96, *SE* = .09; 2: *M* = 4.44, *SE* = .10; *p* < .01), the casual attire (*M* = 4.24, *SE* = .11, *p* < .1) and the formal attire (*M* = 3.33, *SE* = .10, *p* < .01). Although smart attire 2 (men: no tie; women: trousers; *M* = 4.44, *SE* = .10) was preferred over smart attire 1 (men: tie; women: skirt; *M* = 3.96, *SE* = .09, *p* < .01), both smart attires and the casual attire were considered more suitable for dentists than formal attire (suit; *M* = 3.33, *SE* = .10, *p* < .01).

The two-way interaction between target gender and attire, *F*(4, 796) = 45.10, *p* < .001, η^2^p = .19, indicated that participants generally considered men more suitable for dentistry than women; however, this effect was stronger for smart attire 1, *F*(1, 200) = 227.06, *p* < .001, η^2^p = .53; and smart attire 2, *F*(1, 200) = 218.15, *p* < .001, η^2^p = .52 than for professional, *F*(1, 200) = 61.47, *p* < .001, η^2^p = .24; casual, *F*(1, 200) = 8.26, *p* < .01, η^2^p = .04; or formal, *F*(1, 200) = 11.06, *p* < .001, η^2^p = .05.

#### Lawyers

When analyzing the suitability of attire for lawyers, all main effects were significant. Male lawyers (*M* = 4.61, *SE* = .06) were rated as more suitable to the profession than female lawyers (*M* = 4.13, *SE* = .07), *F*(1,199) = 70.37, *p* < .001, η^2^p = .26. The main effect of attire was significant, *F*(4, 796) = 109.55, *p* < .001, η^2^p = .85, and post hoc pairwise comparisons revealed formal attire (Suit; *M* = 6.74, *SE* = .08) to be significantly more suitable for lawyers than both smart attires (1: *M* = 5.84, *SE* = .09; 2: *M* = 5.42, *SE* = .10, *p* < .001) and both casual attires (1: *M* = 1.98, *SE* = .08; 2: *M* = 1.86, *SE* = .08, *p* < .001). Smart attire 1 (men: tie; women: skirt; *M* = 5.84, *SE* = .09) was rated significantly more suitable than smart attire 2 (men: no tie, women: trousers; *M* = 5.42, *SE* = .10, *p* < .001); however, both smart attires were preferred over both casual attire 1 (men: trousers and top; women: skirt and t-shirt; *M* = 1.98, *SE* = .08, *p* < .001) and casual 2 (men and women: jeans and t-shirt; *M* = 1.86, *SE* = .08, *p* < .001). No significant differences were observed between the two casual attires (*p* > .05).

The two-way interaction between target gender and attire, *F*(4, 796) = 52.71, *p* < .001, η^2^p = .21, indicated that participants rated men more suitable to the profession across all attires except casual attire 1, *F*(1, 200) = .60, *p* > .05, η^2^p = .00. The effect was stronger for smart 1, *F*(1, 200) = 104.51, *p* < .001, η^2^p = .34; and formal, *F*(1, 200) = 121.12, *p* < .001, η^2^p = .38, than for smart 2, *F*(1, 200) = 7.25, *p* < .01, η^2^p = .04; and casual 2, *F*(1, 200) = 10.33, *p* < .01, η^2^p = .05.

### Attire and capability

Question B assessed the capability of the model with respect to their profession (dentist or lawyer). Mauchley's test of sphericity yielded a significant effect of attire, D: χ^2^(9) = 133.66, *p* < .001; L: χ^2^(9) = 247.53, *p* < .001, and a significant interaction of Gender × Attire, D: χ^2^(9) = 94.21, *p* < .001; L: χ^2^(9) = 19.17, *p* < .05.

#### Dentists

The 2 × 5 ANOVA was repeated using the same within-subjects variables to investigate participant capability ratings. All main effects were significant. Men (*M* = 5.49, *SE* = .08) were rated as significantly more capable than women (*M* = 4.08, *SE* = .08), *F*(1,199) = 285.52, *p* < .001, η^2^p = .59. Post hoc pairwise comparisons for the main effect of attire, *F*(4,796) = 159.28, *p* < .001, η^2^p = .45, revealed that models in professional attire (white coat; *M* = 6.53, *SE* = .10) were considered more capable than those in both smart attires (1: *M* = 4.46, *SE* = .10; 2: *M* = 4.57, *SE* = .10, *p* < .001) and the casual attire (*M* = 4.49, *SE* = .11, *p* < .001). In addition, all attires were associated with greater capability than the formal attire (suit; *M* = 3.88, *SE* = .11, *p* < .001).

The two-way interaction between target gender and attire, *F*(4, 796) = 31.53, *p* < .001, η^2^p = .14, indicated that participants generally considered men more capable than women; however, this effect was stronger for smart attire 1 (M: tie vs. F: skirt), *F*(1, 200) = 316.68, *p* < .001, η^2^p = .61, and smart attire 2 (M: no tie vs. F: trousers), *F*(1, 200) = 206.90, *p* < .001, η^2^p = .51, than for professional, *F*(1, 200) = 87.16, *p* < .001, η^2^p = .30; casual, *F*(1, 200) = 28.43, *p* < .001, η^2^p = .12; or formal, *F*(1, 200) = 33.57, *p* < .001, η^2^p = .14.

#### Lawyers

Male lawyers (*M* = 4.73, *SE* = .07) were rated as more capable than female lawyers (*M* = 4.32, *SE* = .08), *F*(1,199) = 48.83, *p* < .001, η^2^p = .20. Post hoc pairwise comparisons for the main effect of attire, *F*(4,796) = 859.77, *p* < .001, η^2^p = .81, revealed that models in formal attire (suit; *M* = 6.70, *SE* = .08) were rated as significantly more capable than those adorning both smart attire 1 (M = tie, F = skirt; *M* = 5.85, *SE* = .09, *p* < .001), and smart attire 2 (M = no tie, F = trousers; *M* = 5.43, *SE* = .10, *p* < .001). In turn, formal attire and both smart attires were rated as significantly more capable than casual dress 1 (M = casual trousers, F = casual skirt; *M* = 2.40, *SE* = .09, *p* < .001) and 2 (M and F = jeans and t-shirt; *M* = 2.26, *SE* = .10, *p* < .001). Mean capability ratings for the two variations of casual dress did not significantly differ (*p* > .05).

The two-way interaction between gender and attire, *F*(4,796) = 46.54, *p* < .001, η^2^p = .19, indicated that men were generally rated as more capable than women when adorning smart attires 1, *F*(1,200) = 90.21, *p* < .001, η^2^p = .31; and 2, *F*(1,200) = 12.74, *p* < .001, η^2^p = .06, as well as casual attire 2, *F*(1,200) = 11.42, *p* < .01, η^2^p = .05, but the effect was stronger for formal attire, *F*(1,200) = 111.25, *p* < .001, η^2^p = .36, and no effect was observed for casual attire 1, *F*(1,200) = .65, *p* > .05, η^2^p = .00.

### Attire and ease of talking

Question C assessed how easy the dentists and lawyers looked to talk to, with respect to their profession. Mauchley's test of sphericity yielded a significant effect of attire, D: χ^2^(9) = 131.80, *p* < .001; L: χ^2^ (9) = 353.61, *p* < .001, and a significant interaction of Gender × Attire, D: χ^2^(9) = 60.79, *p* < .001; L: χ^2^(9) = 50.00, *p* < .001.

#### Dentists

The significant main effect of gender indicated that male dentists (*M* = 5.16, *SE* = .08) were rated as easier to talk to than female dentists (*M* = 4.20, *SE* = .08), *F*(1,199) = 112.26, *p* < .001, η^2^p = .36. There was also a main effect of attire, *F*(4,796) = 69.67, *p* < .001, η^2^p = .26, and post hoc Bonferroni correction revealed that professional attire (white coat; *M* = 5.42, *SE* = .11) was rated as significantly easier to talk to than either smart attire (1: *M* = 4.72, *SE* = .09; 2: *M* = 4.79, *SE* = .09, *p* < .001) and the casual attire (*M* = 4.74, *SE* = .10, *p* < .001). All attires were rated as significantly easier to talk to than formal attire (suit; *M* = 3.71, *SE* = .08, *p* < .001). Ratings did not significantly differ between the two variations of smart attire or the casual attire (*p* > .05).

The two-way interaction between target gender and attire, *F*(4, 796) = 6.99, *p* < .001, η^2^p = .03, indicated that men were generally considered easier to talk to than women, but this effect was stronger for smart attire 2 (M = no tie vs. F = trousers), *F*(1, 200) = 98.85, *p* < .001, η^2^p = .33; and casual, *F*(1, 200) = 50.77, *p* < .001, η^2^p = .20, than smart attire 1 (M = tie vs. F = skirt), *F*(1, 200) = 41.49, *p* < .001, η^2^p = .17; professional (white coat), *F*(1, 200) = 24.49, *p* < .001, η^2^p = .11); or the formal suit, *F*(1, 200) = 35.25, *p* < .001, η^2^p = .15.

#### Lawyers

Results highlighted a main effect of target gender: male lawyers (*M* = 4.93, *SE* = .07) were rated as significantly easier to talk to than female lawyers (*M* = 4.45, *SE* = .08), *F*(1,199) = 59.68, *p* < .001, η^2^p = .23. There was also a significant main effect of attire, *F*(4,796) = 35.01, *p* < .001, η^2^p = .15, and post hoc pairwise comparisons revealed lawyers in formal attire (suit; *M* = 5.15, *SE* = .09) and both smart attires (1: *M* = 4.96, *SE* = .08; 2: *M* = 5.04, *SE* = .09) to be rated as significantly easier to talk to than models adorning both casual attires (1: *M* = 4.21, *SE* = .12; 2, *p* < .01; *M* = 4.10, *SE* = .13, *p* < .1). Post hoc comparisons between the two variations of smart attire; and the two variations of casual attire did not reveal significantly different capability ratings (*p* > .05).

The two-way interaction between target gender and attire, *F*(4,796) = 18.64, *p* < .001, η^2^p = .09, indicated that men were rated as easier to talk to than women when wearing formal attire, *F*(1,200) = 91.69, *p* < .001, η^2^p = .31; smart attire 1 (M = tie vs. F = skirt), *F*(1,200) = 68.29, *p* < .001, η^2^p = .26; and casual attire 2 (M = jeans and t-shirt vs. F = jeans and t-shirt), *F*(1,200) = 8.01, *p* < .01, η^2^p = .04, but not when adorning smart attire 2 (M = no tie vs. F = trousers), *F*(1,200) = 3.25, *p* > .05, η^2^p = .02, or casual 1 (M = casual trousers and top vs. F = casual skirt and t-shirt), *F*(1,200) = .01, *p* > .05, η^2^p = .00.

### Attire and perceived friendliness

Question D assessed the friendliness of the dentists and lawyers. Mauchley's test of sphericity yielded a significant effect of attire, D: χ^2^(9) = 151.76, *p* < .001; L: χ^2^ (9) = 372.67, *p* < .001, and a significant interaction of Gender × Attire, D: χ^2^(9) = 56.26, *p* < .001; L: χ^2^ (9) = 39.75, *p* < .001.

#### Dentists

The ANOVA returned a significant main effect for target gender: men (*M* = 5.14, *SE* = .07) were rated as friendlier than women (*M* = 4.23, *SE* = .08), *F*(1,199) = 109.09, *p* < .001, η^2^p = .35; and for attire, *F*(4,796) = 69.33, *p* < .001, η^2^p = .26. Post hoc pairwise comparisons revealed that professional attire (white coat; *M* = 5.28, *SE* = .10) was rated as significantly friendlier than all other attires: smart 1 (M = tie, F = skirt; *M* = 4.77, *SE* = .09) and 2 (M = no tie, F = trousers; *M* = 4.80, *SE* = .09); casual (*M* = 4.89, *SE* = .10); and formal attire (*M* = 3.66, *SE* = .09; *p* < .05). The formal dark suit was rated as significantly less friendly than all other attires (*p* < .001). The mean ratings for two variations of smart attire did not significant differ (*p* > .05) and neither smart attire was considered significantly more friendly than the casual attire (*p* > .05).

The two-way interaction between target gender and attire, *F*(4, 796) = 5.95, *p* < .001, η^2^p = .03, indicated that men were generally rated as friendlier than women, but this effect was stronger for smart attire 2, *F*(1, 200) = 88.14, *p* < .001, η^2^p = .31, and casual attire, *F*(1, 200) = 57.32, *p* < .001, η^2^p = .22, than for smart attire 1, *F*(1, 200) = 28.46, *p* < .001, η^2^p = .13; professional, *F*(1, 200) = 25.50, *p* < .001, η^2^p = .11; or formal attire, *F*(1, 200) = 32.04, *p* < .001, η^2^p = .14.

#### Lawyers

Results highlighted a main effect of target gender: male lawyers (*M* = 4.89, *SE* = .07) were rated as significantly more friendly than female lawyers (*M* = 4.42, *SE* = .08), *F*(1,199) = 57.65, *p* < .001, η^2^p = .23. There was a main effect of attire, *F*(4,796) = 16.00, *p* < .001, η^2^p = .07, and post hoc Bonferroni corrections revealed formal attire (*M* = 4.98, *SE* = .09) and both smart attires (1: *M* = 4.86, *SE* = .08; 2: *M* = 4.89, *SE* = .09) to be significantly more friendly than both casual attires (1: *M* = 4.24, *SE* = .12; 2: *M* = 4.30, *SE* = .13, *p* < .01). However, no significant differences were identified between the mean ratings of the two smart attires, nor between smart attire 1 and formal attire (*p* > .05).

The two-way interaction between target gender and attire, *F*(4, 796) = 16.96, *p* < .001, η^2^p = .08, indicated that men were rated as friendlier than women when adorning smart attire 1 (M = tie vs. F = skirt), *F*(1, 200) = 42.31, *p* < .001, η^2^p = .18; and casual 2 (M = jeans and t-shirt vs. F = jeans and t-shirt), *F*(1, 200) = 25.73, *p* < .001, η^2^p = .11, but the effect was stronger for lawyers wearing formal attire (M = suit vs. F = suit); *F*(1, 200) = 84.62, *p* < .001, η^2^p = .30. No significant gender differences were observed for lawyers wearing smart attire 2 (M = no tie vs. F = trousers), *F*(1, 200) = 1.12, *p* > .05, η^2^p = .01) or casual 1 (M = *F*(1, 200) = .09, *p* > .05, η^2^p = .00).

## Discussion

The purpose of this study was to investigate the influence of a number of attires, varying in formality, on the perceived professionalism of men and women in two occupations—dentists and lawyers—that often involve limited professional–client interface. Previous research suggests that varying levels of formality in attire project various professional characteristics (Barrett & Booth, 1994; Fortenberry et al., 1978; Gledhill et al., 1997; Kwon & Johnson, 1998). The present study tested three hypotheses, two of which were supported. Taken together, the results suggest that the perceived professionalism, including suitability, capability, ease to talk with, and friendliness of professionals in both occupations is significantly influenced by the choice of dressing style worn for work.

Hypothesis 1a, which was that participants would show an absolute preference for male over female professionals and in professional/formal attire (Hypothesis 1b), was supported by the present results. Historically, there has been a clear underrepresentation of women in white-collar male-dominated occupations, which has led to work segregation. The characteristics required of the modern-day professional dentist and lawyer—control, credibility, and decisiveness—are male gender-typed (Bem, 1975; Spence, Helmreich, & Stapp, 1975) and thus gender–role congruence results in individuals often unknowingly thinking of men in these roles. For example, female professionals have been found to be perceived as less competent and more emotionally unstable than their male counterparts, across cultures and time periods, regardless of what they are wearing (Engleman, 1974). Such perceptions are particularly true in male-dominated fields such as medicine and law (Deaux & Emshwiller, 1974; Feldman-Summers & Keisler, 1974; Mischel, 1974). Consequently, when given the option, individuals will choose to seek the expertise of a male dentist and lawyer. Replicating previous research, the present findings suggest a general resistance to female professionals both inside and out of the medical professions.

The preference for professional attire in dentistry (shirt, tie, trousers/skirt, t-shirt, and white coat) supports previous research to suggest a preference for formality in the medical professions and a greater willingness to share personal information with those who wear the recognizable and trusted white coat (Barrett & Booth, 1994; Dunn, Lee, Percelay, & Goldman, 1987; Gherardi et al., 2009; Gjerdingen et al., 1987; Kanzler & Gorsulowsky, 2002; Menahem & Shvartzman, 1998; Rehman et al., 2005). They refute the findings of Lill and Wilkinson (1981) who found that patients prefer doctors to dress in a smart-formal style. They support the study of McKenna et al. (2007), using different stimuli, that patients prefer their dentist to smartly dress and with a white coat. The white coat is a symbolic icon of medical professions (Blumhagen, 1979) and the present findings suggest that its importance can be extended to dentistry, despite it not being prescribed by the U.K. Department of Health, and not forming part of the traditional dentist attire. The present finding lends itself to the suggestion that long-lasting impressions are formed by professional dress in the medical profession, as it does in others (Fortenberry et al., 1978; Kwon & Johnson, 1998; Matsui et al., 1998).

The finding that participants showed an absolute preference for male lawyers in formal dressing style (suit and tie) supports previous research to suggest that conservative clothing symbolizes not only the traditional professional business attire, but also reliability and conveys the individual as authoritative, competent, and able to deliver. Notably, formal attire has been found across a number of contexts to generate an impression of status and power (Fortenberry et al., 1978; Kwon & Johnson, 1998). Given the often intense power-dependency relationship between lawyer and client, the present findings clearly attest to the importance of managing a formal dressing style that conveys power and competence and that, by not adorning such attire, lawyers run the risk of negatively influencing the perceived quality of legal representation.

Hypothesis 2, which was that dentists and lawyers wearing professional and formal attire would be perceived as more suitable and capable in their respective professions than those adorning casual attire (casual skirt/jeans and t-shirt), was supported by the present findings. Models adorning casual attire were rated as the least suitable for the role and the least capable. Male dentists were generally considered more suitable and capable than female dentists and the effect was strongest when adorning smart attire. Similarly, male lawyers were rated as more suitable and capable than female lawyers, especially when dressed in the formal dark suit. In line with previous research, these findings suggest an association between more formal attire and perceived competence in the job role (e.g., Barrett & Booth, 1994; Gherardi et al., 2009).

When compared with smart attire, the professional white coat worn by dentists—rated the most professional—showed reduced target sex differences when compared with other attires. However the most professional dressing style across all four traits for lawyers (the formal suit) showed generally larger sex differences than the casual outfit of jeans and a t-shirt (the least professional attire). This finding was surprising as the white coat has traditionally been a gender-neutral symbol of status and competence that conceals gender-typed biases that are frequently associated with these traits (Gherardi et al., 2009). Albeit less pronounced, the dark formal suit resulted in larger target sex differences across the professionalism traits. There are two potential explanations for this finding. First, jeans and a t-shirt are rarely worn during client-facing meetings and convey laziness, disinterest, and lack of investment in the client. These associations are so pervasive and perceptions of professionalism will be so low that it is almost arbitrary whether it is man or woman adorning this dressing style. However, the traditional business suit and its gender-typed associations with power, status, and confidence automatically result in perceptions of men as significantly more professional in this dressing style. Alternatively, this finding could reflect a limitation of the present study, namely that women's fashions change at a much faster pace than that of men's. The photos used in the present study were adapted from previous research. Taken together, it is possible that participant professionalism ratings were based on the more noticeable outdated dressing style of the female professional than that of the male professional.

Hypothesis 3, which was that professionals wearing casual attire would be perceived as friendlier and as easier to talk to than those adorning professional or formal attire, was not supported by the present findings. Results suggested that dentists in professional attire were perceived as easier to talk to and as friendlier, whereas those adorning formal attire were considered the least easy to talk with and the least friendly. The finding that men are perceived as easier to talk to and as friendlier than women does not lend support to previous research highlighting women's gender-typed communal, sensitive, and caring interpersonal style (Eagly & Koenig, 2008; Eagly & Wood, 2000). A similar pattern of results were observed in the lawyer condition. Men were rated as significantly friendlier and easier to talk to than women, as were those wearing a formal dressing style. When compared with all other attires, models adorning casual attire were considered the least friendly and as less easy to talk to. These findings refute earlier research, which has identified a casual dressing style as friendlier, more gentle and more approachable (Barrett & Booth, 1994; Cardon & Okoro, 2009; Skorupski & Rea, 2006), as well as that suggesting professional characteristics are communicated along a continuum of formal to casual workplace attire (Cardon & Okoro, 2009).

There are two potential explanations for this finding. It is possible that perceptions of friendliness and ease of talking with professionals are mediated by perceptions of suitability and capability. If clients do not consider the professional to be suitably competent in their role, it is less likely that information will be divulged or that a long-term professional–client relationship will develop. An alternative explanation is that, while women are stereotypically perceived as more interpersonal and caring, it is possible that the female professionals in the present study were perceived as less interpersonally friendly than other women because of their perseverance and success in a male-dominated profession. Thus, it is possible that, when judging approachability and ease of communication with female professionals, participants compared the female targets with other women who have not taken these career paths, rather than with male professionals. It is also possible that participants interpreted these two communication traits in relation to the job role rather than as individual traits of men and women.

Taken together, these findings suggest that choosing a more casual dressing style in the workplace is unlikely to be an effective tactic when the aim was to increase client disclosure and ease of communication. Dentists and lawyers who choose to wear white apparel and a formal suit, respectively, are more likely to build a long-term relationship with clients and gather the information necessary for desired health/legal outcomes. Considering the additional pressures placed on female professionals when developing these traits, the choice of attire may be critical to the mutually shared interests of professional and client.

In conclusion, this study found that when presented with a photograph of an unknown dentist and lawyer, before the development of a relationship, individuals prefer the white coat or formal dark suit over more informal dressing styles. Company dress code guidelines may not necessarily prescribe a white coat or dark suit during face-to-face professional–client interactions; however, the present findings suggest that clients prefer these dressing styles, and that they may provide an air of credibility when providing dental/legal advice.

In terms of limitations, the authors acknowledge that investigation of preferences from photographs does not account for the many other factors involved in professional–client communication such as physical demeanor, charisma, or empathy that may be used to infer traits such as capability, friendliness, and approachability. This caution should be exercised when extrapolating the present findings to real face-to-face encounters.

Additionally, no effort was made to control for the perceived likeability or competence of the target professionals, outside of their dentist/lawyer roles. A replication of the present study would benefit from controlling for these potential confounds so as to be confident that target sex differences in professionalism are the result of genuine participant preferences for male dentists and lawyers rather than a preference for enhanced attractiveness and/or competence. To date no studies in this area have made a very serious attempt to control for all the potential confounds in the use of photographic models. We made some effort to control for age and attractiveness, but accept that other confounds were occurring. In addition, it is important that researchers keep up to date with their models as hairstyle and casual wear can easily go out of fashion.

Lastly, self-report data do not provide an overall gauge of dentist and lawyer visitations, but rather an intention to seek health or legal assistance. Equally, studies of this kind cannot take into consideration other factors like the professional voice or accent, which could have a very dramatic effect on any impression given with the attire. Future studies may well consider using video stimuli rather than photographs or even live models to see to what extent clothes alone effect impressions and how long they last when supplemented by other data.

Its limitations notwithstanding, this study has provided insight into the differential perceptions of professionalism of dentists and lawyers as a function of their dressing style. Findings have potentially important implications for newly qualified individuals. Specifically, newly qualified dentists and lawyers may consider wearing more professional or formal attire during their patient/client interactions. This is likely to favorably influence their perceived capability and friendliness, which in turn will positively influence perceptions of trust and facilitate sharing of personal information. In addition, professionals should develop an awareness of the various professional characteristics associated with dressing more formally and more casually. Simultaneously, an education program informing patients/clients of the reasons behind wearing different attires will serve to increase perceptions of professionalism in the individual professional as well as confidence in the profession. This is particularly important if the attire worn to work influences adherence to dental care regimen and positive legal outcomes.

## References

[b1] Barrett TG, Booth IW (1994). Sartorial eloquence: Does it exist in the paediatrician-patient relationship?. British Medical Journal.

[b2] Becker H, Greer B, Hughes E, Strauss A (1961). Boys in white: Student culture in a medical school.

[b3] Bem SL (1975). Sex role adaptability: One consequence of psychological androgyny. Journal of Personality and Social Psychology.

[b4] Bersheid E, Gangestad S (1982). The social psychological implications of facial physical attractiveness. Clinics in Plastic Surgery.

[b5] Bixler S, Scherrer Dugan L (2000). 5 steps to professional presence: How to project confidence, competence, and credibility at work.

[b6] Blumhagen DW (1979). The doctor's white coat: The image of the physician in modern America. Annals of Internal Medicine.

[b7] Bond L, Clamp P, Gray K, Van Dam V (2010). Patients' perceptions of doctors' clothing. The Journal of Laryngology and Otology.

[b8] Brosky ME, Keefer OA, Hodges JS, Pesun IJ, Cook G (2003). Patient perceptions of professionalism in dentistry. Journal of Dental Education.

[b9] Cardon PW, Okoro EA (2009). Professional characteristics communicated by formal versus casual workplace attire. Business Communication Quarterly.

[b10] Cha A, Hecht B, Nelson K, Hopkins M (2004). Resident physician attire. American Journal of Obstetrics and Gynaecology.

[b11] Cho E, Grover L (1978). Looking terrific: Express yourself through the language of clothing.

[b12] Colt HG, Solot JA (1989). Attitudes of patients and physicians regarding physician dress and demeanor in the emergency department. Annals of Emergency Medicine.

[b13] Deaux K, Emshwiller T (1974). Explanation of successful performance on sex-linked tasks: What is skill for the male is luck for the female. Journal of Personality and Social Psychology.

[b14] Douse J, Derrett-Smith E, Dheda K, Dilworth JP (2004). Should doctors wear white coats?. Postgraduate Medical Journal.

[b15] Dunn J, Lee T, Percelay J, Goldman L (1987). Patient and house officer attitudes on physician attire and etiquette. Journal of the American Medical Association.

[b16] Eagly AH, Borgida E, Fiske ST, Koenig AM (2008). Gender prejudice: On the risks of occupying incongruent gender roles. Beyond common sense: Psychological science in the courtroom.

[b17] Eagly AH, Wood W, Eckes T, Trautner HM (2000). Social role theory of sex differences and similarities: A current appraisal. Developmental social psychology of gender.

[b18] Engleman EG (1974). Attitudes toward women physicians: A study of 500 clinic patients. The Western Journal of Medicine.

[b20] Feldman-Summers S, Keisler SB (1974). Those who are number two try harder: The effect of sex on attributions of causality. Journal of Personality and Social Psychology.

[b19] Fischer R, Hansen C, Hunter R, Veloski J (2007). Does physician attire influence patient satisfaction in an outpatient obstetrics and gynaecology setting?. American Journal of Obstetrics and Gynaecology.

[b21] Fortenberry JH, MacLean J, Morris P, O'Connell M (1978). Mode of dress as a perceptual cue to deference. The Journal of Social Psychology.

[b22] Gherardi G, Cameron J, West A, Crossley M (2009). Are we dressed to impress? A descriptive survey assessing patients' preference of doctors' attire in the hospital setting. Clinical Medicine.

[b23] Gjerdingen DK, Simpson DE (1989). Physicians' attitudes about their professional appearance. The Family Practice Research Journal.

[b24] Gjerdingen DK, Simpson DE, Titus SL (1987). Patients' and physicians' attitudes regarding the physician's professional appearance. Archives of Internal Medicine.

[b25] Gledhill JA, Warner JP, King M (1997). Psychiatrists and their patients: Views on forms of dress and address. The British Journal of Psychiatry.

[b26] Goffman E (1959). The presentation of self in everyday life.

[b27] Gonzalez Del Rey JA, Paul RI (1995). Preferences of parents for pediatric emergency physicians' attire. Pediatric Emergency Care.

[b28] Gooden BR, Smith MJ, Tattersall SJ, Stockler MR (2001). Hospitalised patients' views on doctors and white coats. The Medical Journal of Australia.

[b29] Harnett PR (2001). Should doctors wear white coats?. The Medical Journal of Australia.

[b30] Hennessy N, Harrison DA, Aitkenhead AR (1993). The effect of the anaesthetist's attire on patient attitudes: The influence of dress on patient perception of the anaesthetist's prestige. Anaesthesia.

[b31] Hippocrates, Jones WHS (1923). Hippocrates. Hippocrates.

[b32] Ikusaka M, Kamegai M, Sunaga T, Narita N, Kobayashi H, Yonenami K (1999). Patients' attitudes towards consultations by a physician without a white coat in Japan. Internal Medicine (Tokyo, Japan).

[b33] Kalisch BJ, Kalisch PA (1985). Dressing for success. The American Journal of Nursing.

[b34] Kanzler MH, Gorsulowsky DC (2002). Patients' attitudes regarding physical characteristics of medical care providers in dermatologic practices. Archives of Dermatology.

[b35] Kwon YH, Johnson HJ (1998). College students' perceptions of occupational attributes based on formality of business attire. Perceptual and Motor Skills.

[b36] LaSala KB, Nelson J (2005). What contributes to professionalism?. Medsurgical Nursing.

[b37] Levitt M (1981). The executive look: How to get it—How to keep it.

[b38] Lill MM, Wilkinson TJ (2005). Judging a book by its cover: Descriptive survey of patients' preferences for doctors' appearance and mode of address. British Medical Journal.

[b39] Lurie A (1981). The language of clothes.

[b40] Matsui D, Cho M, Rieder MJ (1998). Physicians' attire as perceived by young children and their parents: The myth of the white coat syndrome. Pediatric Emergency Care.

[b41] McKenna G, Lillywhite G, Maini N (2007). Patient preferences for dental clinical attire. British Dental Journal.

[b42] McKinstry B, Wang J (1991). Putting on the style: What patients think of the way their doctor dresses. The British Journal of General Practice.

[b43] Menahem S, Shvartzman P (1998). Is our appearance important to patients?. Family Practice.

[b44] Mischel H (1974). Sex bias in the evaluation of professional achievements. Journal of Educational Psychology.

[b45] Molloy J (1975). Dress for success.

[b46] Molloy J (1977). The Woman's dress for success.

[b47] Niederhauser A, Turner M, Chauhan S, Magann E, Morrison J (2009). Physician attire in the military setting. Military Medicine.

[b48] Rafaeli A, Pratt MG (1993). Tailored meanings: On the meaning and impact of organizational dress. Academy of Management Review.

[b49] Rehman SU, Nietert PJ, Cope DW, Kilpatrick AO (2005). What to wear today? Effect of doctor's attire on the trust and confidence of patients. The American Journal of Medicine.

[b50] Rose A (1962). Human behavior and social process.

[b51] Simmel G (1971). On individuality and social forms: Selected writings.

[b52] Singer JE, Brush CA, Lublin SC (1965). Some aspects of deindividuation: Identification and conformity. Journal of Experimental Social Psychology.

[b53] Skorupski V, Rea R (2006). Patients' perceptions of today's nursing attire. The Journal of Nursing Administration.

[b54] Solomon M (1986). Dress for effect. Psychology Today.

[b55] Solomon M (1987). Standard issue. Psychology Today.

[b56] Solomon MR, Douglas SP (1983). Diversity in product symbolism: The case of female executive clothing. Psychology and Marketing.

[b57] Spence JT, Helmreich R, Stapp J (1975). Ratings of self and peers on sex role attributes and their relation to self-esteem and conceptions of masculinity and femininity. Journal of Personality and Social Psychology.

[b58] Stone GP, Marshall RA (1962). Appearance and the self. Human behaviour and social processes: An interactionist approach.

[b59] Swift G, Zachariah M, Casy PR (2000). A rose by any other name: Psychiatric outpatients' views on dress and address. Irish Journal of Psychological Medicine.

[b60] Taylor PG (1987). Does dress influence how parents first perceive house staff competence?. Archives of Pediatrics and Adolescent Medicine.

[b61] Veblen T (1899). The theory of the leisure class: An economic study of institutions.

